# The mental health crisis of expectant women in the UK: effects of the COVID-19 pandemic on prenatal mental health, antenatal attachment and social support

**DOI:** 10.1186/s12884-022-04387-7

**Published:** 2022-01-26

**Authors:** Maria Laura Filippetti, Alasdair D. F. Clarke, Silvia Rigato

**Affiliations:** Centre for Brain Science, Department of Psychology, University of Essex, Colchester, UK

**Keywords:** Pregnancy, Maternal mental health, Antenatal attachment, Social support, Prenatal trauma

## Abstract

**Background:**

Pregnancy has been shown to be times in a woman’s life particularly prone to mental health issues, however a substantial percentage of mothers report subclinical perinatal mental health symptoms that go undetected. Experiences of prenatal trauma, such as the COVID-19 pandemic, may exacerbate vulnerability to negative health outcomes for pregnant women and their infants. We aimed to examine the role of: 1) anxiety, depression, and stress related to COVID-19 in predicting the quality of antenatal attachment; 2) perceived social support and COVID-19 appraisal in predicting maternal anxiety and depression.

**Methods:**

A sample of 150 UK expectant women were surveyed during the COVID-19 pandemic. Questions included demographics, pregnancy details, and COVID-19 appraisal. Validated measures were used to collect self-reported maternal antenatal attachment (MAAS), symptoms of anxiety (STAI), depression (BDI-II), and stress related to the psychological impact of COVID-19 (IES-r).

**Results:**

We found that the pandemic has affected UK expectant mothers’ mental health by increasing prevalence of depression (47%), anxiety (60%) and stress related to the psychological impact of COVID-19 (40%). Women for whom COVID-19 had a higher psychological impact were more likely to suffer from depressive (95% HDPI = [0.04, 0.39]) and anxiety symptoms (95% HPDI = [0.40, 0.69]). High depressive symptoms were associated with reduced attachment to the unborn baby (95% HPDI [-0.46, -0.1]). Whilst women who appraised the impact of COVID-19 to be more negative showed higher levels of anxiety (HPDI = [0.15, 0.46]), higher social support acted as a protective factor and was associated with lower anxiety (95% HPDI = [-0.52, -0.21]).

**Conclusions:**

The current findings demonstrate that direct experience of prenatal trauma, such as the one experienced during the COVID-19 pandemic, significantly amplifies mothers’ vulnerability to mental health symptoms and impairs the formation of a positive relationship with their unborn baby. Health services should prioritise interventions strategies aimed at fostering support for pregnant women.

**Supplementary Information:**

The online version contains supplementary material available at 10.1186/s12884-022-04387-7.

## Background

Research shows that in times of natural disasters, e.g. pandemics, there is an increased risk in mental health issues such as Post-Traumatic Stress Disorder (PTSD), depression, and anxiety [1]. Among the most recent natural disasters, the Coronavirus disease-19 (COVID-19) has led to a global health crisis which has resulted in unprecedented changes in the way we live. In particular, research is now gaining a better understanding of the impact of COVID-19 on psychological outcomes. Specifically, the severe disruptions to education, employment, healthcare, leisure activities, and social events due to the implementation of physical distancing measures may have negatively impacted mental health.

Life changing events may render individuals particularly vulnerable to the impact of disasters. In particular, pregnancy has been extensively shown to be times in a woman’s life especially prone to mental health issues [2–4]. Among psychiatry disorders, the prevalence of depression and anxiety is especially high during pregnancy [5, 6]. However, a substantial percentage of mothers report subclinical perinatal depressive symptoms that go undetected. Critically, while maternal mental health difficulties that classify as subclinical have received little attention, there is some evidence that these can already and significantly affect mothers and their infants [7, 8]. It has been documented that up to 40% of women experiences sub-clinical perinatal depressive symptoms that are likely to remain undetected by practitioners, yet have similar adverse long-term impacts as the more severe symptoms associated with clinical maternal depression [7, 9].

Maternal mental health difficulties, such as depressive or anxiety symptoms, can also have an immediate impact, by interfering and impairing the formation of a positive relationship with the fetus [10]. Maternal antenatal attachment is a multi-dimensional construct that includes maternal thoughts, behaviors, emotions and attitudes towards the unborn child [11]. Previous studies have found that women with higher levels of depression have lower levels of maternal antenatal attachment (e.g. [12, 13], suggesting that maternal mood negatively impacts on the development of an early relationship between a mother and her child. However, a recent systematic review [14] reported mixed results suggesting that the association between depression and maternal antenatal attachment might be more complex than previously thought. The link between maternal anxiety and maternal antenatal attachment is also unclear, although in this case methodological discrepancies might account for the controversial results observed. In particular, while studies which used the Maternal Antenatal Attachment Scale (MAAS [15]; found that higher anxiety was associated with lower MAAS-quality (e.g. [13, 16, 17], no association was found between maternal antenatal attachment and pregnancy related anxiety when using the Maternal Foetal Attachment Scale (MFAS [18];) [19, 20]. Altogether, the available evidence suggests that the relationship between maternal mental health and antenatal attachment deserves further scientific attention.

There exists also evidence that factors from contextual domains, i.e. social support, are related to maternal antenatal attachment [21]. While a meta-analysis of 183 studies of maternal antenatal attachment found a small significant effect of social support on attachment to the fetus [21], other evidence reports no relationship in specific populations of mothers (e.g., adolescents, high-risk pregnant women, etc.) (for a review see [22]. A recent study by Hopkins et al. [23], however, suggests that social support may have both a direct effect on maternal antenatal attachment and also mediate the relationship between maternal anxiety and attachment to her unborn baby. These mixed results overall point towards the complexity of these relationships and suggest that further investigation is warranted.

In the present study, we sought to examine the effects of the COVID-19 pandemic on prenatal mental health, antenatal attachment and social support. According to the intergenerational transition hypothesis (e.g. [24]), direct experience of trauma can impact on the caregiver-infant relationship. Given that prenatal trauma heightens the risk of clinically significant antenatal mental health symptoms [25], we can hypothesise that direct experience of prenatal trauma, such as the one experienced during the COVID-19 pandemic, can affect maternal antenatal attachment by amplifying vulnerability to mental health symptoms. The first aim of the present study was thus to examine whether mental health symptoms of anxiety and depression, as well as stress response due to COVID-19, are predictive of maternal antenatal attachment to the fetus – as measured via the MAAS. Evidence suggests that maternal mental health may be more related to the quality rather than the intensity of antenatal attachment [13, 26]. Therefore, for the purpose of the current study we focused only on the quality subscale of the MAAS.

Recent evidence shows that higher social support is associated with lower mental health symptoms, thus suggesting that support may act as a protective factor, particularly for those who appraise the impact of COVID-19 to be more negative [27]. In line with the buffering model [28], we hypothesised that social support may protect against the negative effects of traumatic events by changing their appraisal. Therefore, our second aim was to investigate whether perceived social support and COVID-19 appraisal predicted mental health symptoms of anxiety and depression.

## Methods

### Participants

Participants were 150 pregnant individuals who took part in the Wellbeing During Pregnancy Study. Online surveys (via Qualtrics) were completed between April 2020 and January 2021. Pregnant individuals were recruited through social media (Facebook and Instagram) advertisements and word of mouth. Participant inclusion criteria were: Over 18 years of age, UK resident, English speaking, and pregnant. At the time of the survey, participants had an average age of 31.06 years (SD = 4.64) and had spent an average of 16.55 years in education (SD = 4.50). The participants’ ethnic background was predominantly white (93%). The majority of participants were married or cohabiting (95%). Twelve percent of participants completed the online survey whilst they were in their first trimester of pregnancy, 40% were in their second trimester, and 48% were in their third trimester of pregnancy.

An additional dataset (*N* = 75) from unpublished online research conducted before the COVID-19 pandemic was used to compare means of the BDI-II scores collected in the current study. The mean age of these participants we 32.71 years (SD = 3.83) and had spent an average of 18.1 years in education (SD = 2.64). The participants’ ethnic background was predominantly white (87%), and the majority of them was married or cohabiting (76%). Further details of the data and additional analysis are presented in Supplementary Material.

### Sample size calculation

To explore our ability to detect effects in the data, we carried out a simulation approach in which we generated depression and anxiety scores from a multivariate Gaussian distribution (Mean = 0 and covariance matrix [1, 0.3; 0.3, 1]). We then generated scores for maternal antenatal attachment under the assumption that a change of 1 for either depression or anxiety was associated with a change of - 0.25 in maternal antenatal attachment. The final step in our data generation process was to add Gaussian noise (Mean = 0, SD = 1) to the maternal antenatal attachment. For an example of the simulated data please see Fig. 1S in the Supplementary Material. This data simulation process was repeated a number of times for different sample sizes. For each simulated dataset we carried out our planned analysis and computed the 95% HPDI. The results of this procedure suggest that with *N* = 140 we should expect a 95% HPDI of [-0.42, -0.07]. In other words, we can reliably detect a simulated effect of - 0.25. For comparison, the effects discussed in the Results section are typically at least +/- 0.3, suggesting that the effects detected by our study are reliable.

### Design and procedure

Before taking part in the study, participants were provided with an electronic information sheet and consent form and were asked to tick a box to confirm consent. At the end of the survey, an electronic debrief with signposting to relevant support information was included. Participants had the option to enter a draw to win one of three £20 vouchers.

Pregnancy-related demographic questions were asked at the beginning of the survey (see Table 1 for summary of participant characteristics). The survey also included questions about the effect that the COVID-19 pandemic has had on the participant’s life, and on their relationships. These questions included the support that participants perceived in relation to their partner, their family and friends, and the healthcare system (e.g. their midwife).

**Table 1:** Participants and COVID-19 characteristics

Participant characteristic	Value	Pregnancy information	Value
*Age (mean year ± SD)*	31.06 (4.64)	High risk pregnancy	43 (29)
*Years in education (mean year ± SD)*	16.55 (4.50)	Low risk pregnancy	107 (72)
*Ethnicity (N/%)*			
White	140 (93)	*Mental health issues before pregnancy (N/%)*	
Black/African/Caribbean/Black British	4 (3)	Yes	85 (57)
Asian/Asian British	1 (1)	No	59 (40)
Mixed/Multiple Ethnic Group	3 (2)	Not sure	6 (4)
Arab	2 (1)	*Mental health issues during pregnancy before COVID-19 (N/%)*	
Other	0 (0)	Yes	49 (33)
Prefer not to answer	0 (0)	No	98 (66)
*Current relationship (N/%)*		Not sure	3 (2)
Married/civil partnership	83 (55)	*Mental health issues during pregnancy since COVID-19 (N/%)*	
Single	4 (3)	Yes	68 (46)
Cohabiting	61 (41)	No	79 (53)
Separated/divorced	1 (1)	Not sure	3 (2)
Widowed	1 (1)	*Suspected COVID-19 (with or without test results)* (*N/%)*	
Prefer not to answer	0 (0)	Yes	20 (15)
*Household size (including participant) (N/%)*		No	112 (85)
1 person	2 (2)	Not sure	0 (0)
2 people	72 (56)	*Partner suspected COVID-19(with or without test results)* (*N/%)*	
3 people	40 (31)	Yes	17 (13)
4 people	19 (15)	No	115 (87)
5 or more people	3 (2)	Not sure	0 (0)
*Access to outdoor space (N/%)*		*Significant person suspected COVID-19 (with or without test results)* (*N/%)*	
Yes	124 (97)	Yes	28 (21)
No	4 (3)	No	83 (63)
		Not sure	21 (16)

### Validated measures

#### Beck depression inventory, 2^nd^ edition

Depressed mood was assessed using the self-report questionnaire Beck Depression Inventory, 2^nd^ Edition (BDI-II [29];. The BDI-II includes 21 questions about depressive symptoms experienced in the past week [30]. Each question asks to what extent a certain state does or does not apply to the respondent (e.g., Sadness; response options: 0 – I do not feel sad, 1 – I feel sad much of the time, 2 – I am sad all the time, 3 – I am so sad or unhappy that I can’t stand it). A higher score indicates a higher level of depressive symptoms. A score between 0 and 13 is classified as “minimal depression”; a score between 14 and 19 is classified as “mild depression”; a score between 20 and 28 is classified as “moderate depression”; and a score between 29 and 63 is classified as “severe depression” [29]. In the current study, Cronbach’s alpha for BDI-II scores was .89.

#### State-trait anxiety inventory

Anxiety symptoms were assessed using the State-Trait Anxiety Inventory (STAI) [31]. The questionnaire includes 20 items that measure current feeling of anxiety (state anxiety; STAI-State) and 20 items that measure how a person generally feels (trait anxiety; STAI-Trait). Participants rated their symptoms based on a 4-point scale (from “not at all” to “very much so”). Scores of 40 and above are considered clinically relevant symptoms of anxiety during pregnancy [32–34]. In the current study, Cronbach’s alpha for STAI-State was .91 and .94 for STAI-Trait. Given the evidence of the widespread impact of subclinical maternal mental health difficulties that are not necessarily linked to stable personality traits [33], in the present study we focused our investigation on the effects of transient reactions to adverse events, rather than more stable personality features. Therefore, for the main analyses of the study we prioritised the role of state anxiety over trait anxiety.

#### Maternal antenatal attachment scale

Maternal antenatal attachment was assessed using the Maternal Antenatal Attachment Scale (MAAS) [15]. This self-report questionnaire consists of 19 items that measure the quality of mother’s affective experiences towards the fetus (“quality of attachment” subscale) and the intensity of preoccupation with the fetus (“time spent in attachment mode” subscale). Higher scores indicate higher attachment. Participants rated their agreement with the items on a 5-point scale. Cronbach’s alpha for the quality of attachment subscale was .73, and .71 for the time spent in attachment mode subscale.

#### Revised impact of event scale

The Revised Impact of Event Scale (IES-r) questionnaire is a self-report measure of stress reactions after traumatic event. The revised version has seven additional questions and a scoring range between 0 and 88 [35]. In the present study, the IES-r was used to measure stress related to the psychological impact of the COVID-19 pandemic. The IES-r questionnaire comprises of two subscales (i.e. intrusive and avoidance). Participants rated their symptoms using a 5-point scale (from “not at all” to “often”). An overall IES score of ⩾24 indicates that post-traumatic stress disorder is of clinical concern [35]. In the current study, a score of ⩾24 was used to indicate moderate-to-severe stress response experienced by pregnant women amid the COVID-19 pandemic (“psychological impact of COVID-19” henceforth). Cronbach’s alpha was .91.

### COVID-19 specific items

During the survey, participants were asked specific questions in relation to the impact of COVID-19 on their relationships and lives [36]. These items specifically referred to the current time period (since the most recent UK lockdown had started). The first item, which we labelled “COVID-19 appraisal” asked “How badly do you think that you will be affected by the global effects of the COVID-19 pandemic (e.g. reduced capacity of health care systems and global financial issues)?”, with response options on a 5-point scale (from “not at all affected” to “severely affected”). The next series of items related to the perceived social support during the pandemic. Specifically, over three questions we asked “How well supported do you feel by your [1. spouse/partner; 2. family/friends; 3. healthcare professionals/midwife] during this time?”. Participants rated their perceived support on a 5-point scale (from “I do not feel supported at all” to “extremely”).

## Results

### Statistical analysis

Descriptive analyses were conducted to identify prevalence rates of depression (BDI-II), anxiety (STAI-State and STAI-Trait), and psychological impact of COVID-19 (IES-r). Bivariate correlations were conducted to identify relationships between variables to inform inferential statistics. We conducted bivariate correlations between the key demographic and pregnancy information (e.g. pregnancy trimester, mental health history) and the MAAS quality of attachment score to determine if any of these variables contributed to the outcome measure. There were no significant correlations between the MAAS quality of attachment score and participant age and years in education. *T*-test comparing women in their first, second and third trimester did not reveal any significant difference between these groups on the MAAS quality of attachment score (first vs second trimester, *t*(22.82) = -1.308, *p* = .204; second vs third trimester, *t*(110.55) = -.191, *p* = .849; first vs third trimester, *t* (22.89) = -1.233, *p* = .231). A *t*-test comparing women with high- and low-risk pregnancies did not reveal any significant differences between these two groups on the MAAS quality of attachment score, *t*(56.63) = –1.746, *p* = .086.

To investigate the relationship between our variables of interest we carried out a series of Bayesian linear models using the Bayesian Regression Models using Stan (brms) [37] package in R v4.0. Variables were centered and scaled, and the models were fit using weakly informative priors (for full details, including a prior predictive check, please see Open Science Framework “OSF” link in Data Statement). After fitting the model to the data, we examined the estimated posterior distributions to see which predictors appeared to be playing a major role. We summarized these distributions by calculating the 95% HDPI (highest density probability interval). The HDPI is the smallest interval such that the probability, given our data, of the “true” value falling within the interval is 95%.

### Prevalence of prenatal mental health issues

Seventy-three out of the 137 women that completed the BDI-II (53%) reported minimal depressive symptoms (scored 13 or below). However, 64 women reported a score > 13 on the BDI-II which indicates the presence of mild or above depressive symptomatology. Specifically, 19% of the sample reported mild depression (scores 14-19), 20% reported moderate depression (scores 20-28), and 7% of participants reported high depression (scored 29 or above). In relation to anxiety symptoms, 60% of participants (90 out of 150 women that completed the STAI-State) had scores above the cut-off based on state anxiety scores (scores of 40 and above) and 62% of participants (90 out of 145 women that completed the STAI-Trait) had scores above the cut-off based on trait anxiety scores. Finally, out of the 126 women that completed the IES-r, 50 participants (40%) reported symptoms associated with post-traumatic stress disorder (a score of ⩾24), suggesting that that the COVID-19 pandemic had a moderate-to-severe psychological impact on their lives.

We also ran additional analysis to compare the depression scores in the current study with data from unpublished research conducted by the senior member of the authorship team before the COVID-19 pandemic. These analyses show that depression scores in the group of women expecting a baby during the second lockdown were significantly higher than the depression scores of women that were pregnant before the COVID-19 pandemic. Statistical details of these analyses are included in Supplementary Material.

### Bayesian modelling

**Fig. 1:**
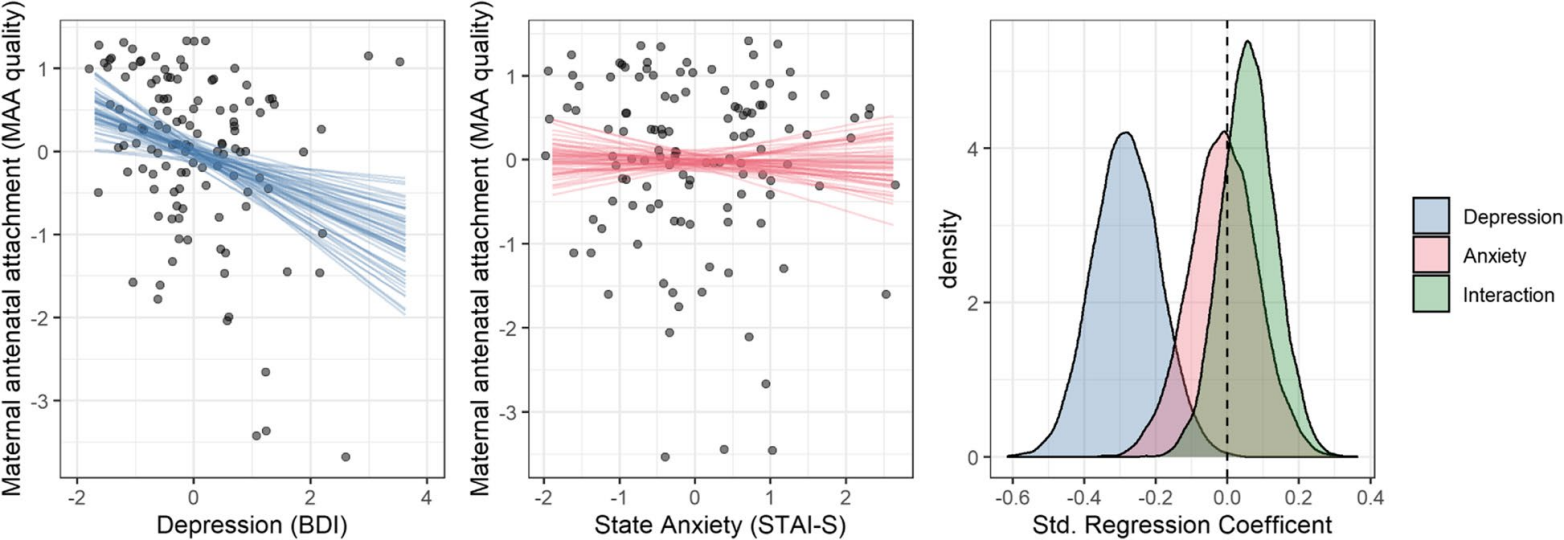
(left) The effect of depression on the quality of maternal antenatal attachment. Each dot indicates a participant while the straight lines show samples from our model’s marginal posterior distribution (while STAI-S was set to 0). (middle) The effect of state anxiety on maternal antenatal attachment. (right) The posterior estimates from our Bayesian linear model

To investigate our first hypothesis, we examined whether STAI-State (anxiety henceforth) and BDI-II (depression henceforth) scores were predictive of the quality of maternal antenatal attachment. As highlighted in Fig. 1, while there is a clear association between depression and quality of attachment, there is little evidence of a correlation with anxiety. To further investigate the relationship among these variables, we carried out Bayesian linear regression to estimate the effect of both anxiety and depression variables, and their interaction, on quality of maternal antenatal attachment. The estimates for the regression slopes are shown in Fig. 1. The analysis shows that, while we can see that depression predicts quality of attachment (95% HPDI [-0.46, -0.1]), neither anxiety (HPDI [-0.20, 0.17]) or the interaction (HPDI [-0.09, 0.21]) appear to have an effect.

**Fig. 2:**
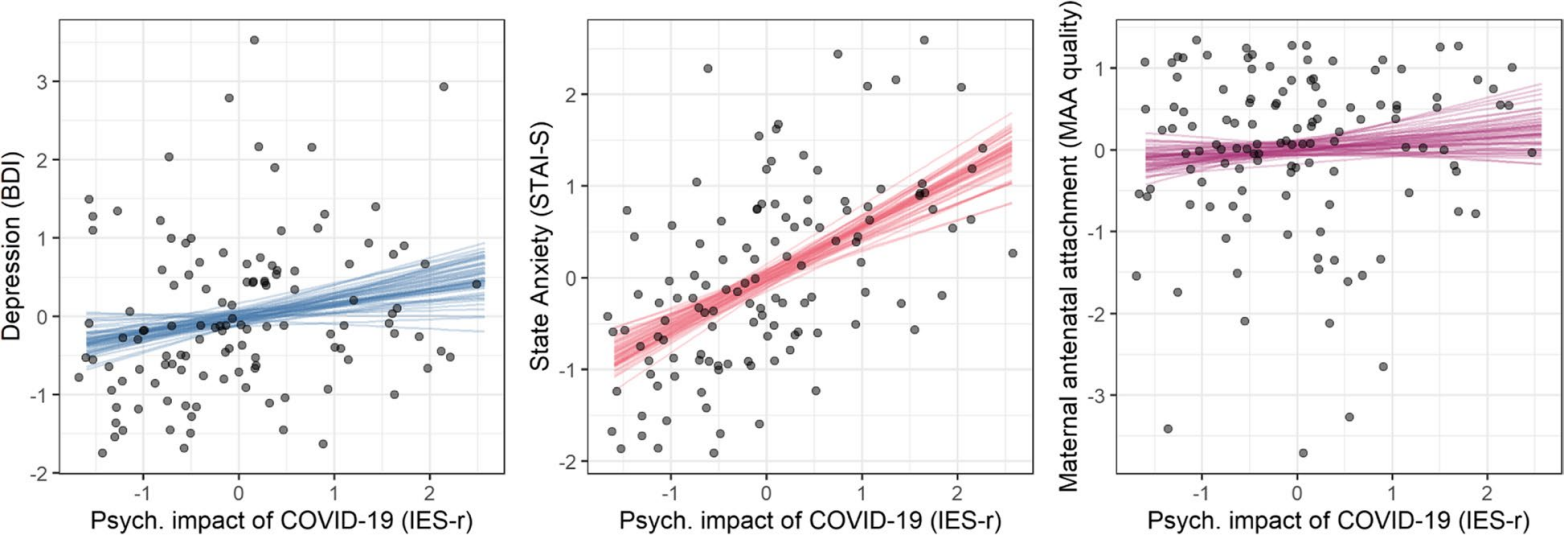
The psychological impact of COVID-19 on (left) depression; (middle) anxiety; and (right) quality of maternal antenatal attachment. Each dot indicates a participant while the straight lines show samples from our model’s marginal posterior distribution

**Fig. 3:**
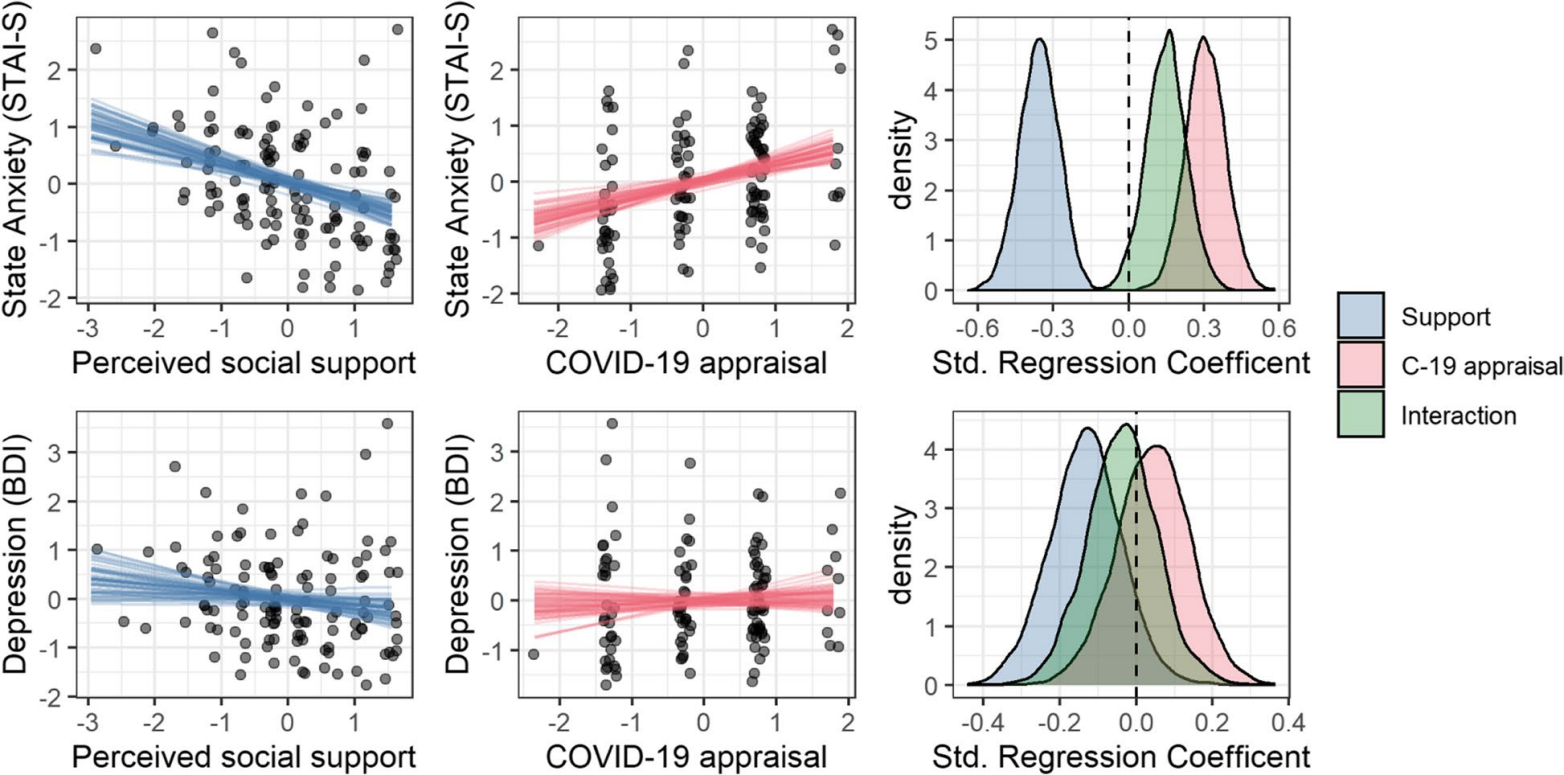
The effect of perceived social support and COVID-19 appraisal on (top row) anxiety and (bottom) depression scores. Each point represents a participant while the lines indicate samples from our model’s marginal posterior distribution. (left col.) The effect of social support on anxiety, for the average value of COVID-19 appraisal. (middle col.) The effect of COVID-19 appraisal on anxiety, for the average value of social support. (right col.) The posterior estimates from our Bayesian linear model

Next, we aimed to investigate the potential effect of the psychological impact of the COVID-19 pandemic (see Fig. 3). We fitted a separate linear model for the effect of IES-r on i) depression, ii) anxiety, and iii) quality of maternal antenatal attachment (See Fig. 2).

We found that the psychological impact of COVID-19 increased both depression (95% HDPI = [0.04, 0.39]) and anxiety (95% HPDI = [0.40, 0.69]) symptoms. The evidence for a direct effect on the quality of maternal attachment is more mixed (95% HDPI = [-0.10, 0.25]), given that the estimated model fits are not reliably < 0.

Our second hypothesis aimed to test the effect of perceived social support and COVID-19 appraisal on maternal anxiety and depression. We modelled both relationships independently, allowing for a potential interaction between the predictors. The modelling results are displayed in Fig. 3 and show that, while anxiety was sensitive to both predictors (social support: 95% HPDI = [-0.52, -0.21], COVID-19 appraisal: HPDI = [0.15, 0.46], interaction: HPDI = [-0.01, 0.31]), neither variable had clear impact on depression (social support: 95% HPDI = [-0.32, 0.05], COVID-19 appraisal: HPDI = [-0.15, 0.23], interaction: HPDI = [-0.22, 0.14]).

## Discussion

With this study, we set out to investigate prenatal maternal wellbeing during the COVID-19 pandemic. The aim of our investigation was twofold; 1) to examine the role of mental health symptoms of anxiety, depression and stress reaction due to COVID-19 in predicting the quality of maternal antenatal attachment to the fetus; 2) to examine the role of perceived social support and COVID-19 appraisal in predicting maternal anxiety and depression. According to the evidence that prenatal trauma heightens the risk of clinically significant antenatal mental health symptoms [25], we expected direct experience of prenatal trauma, i.e. COVID-19 pandemic, to affect maternal antenatal attachment by amplifying vulnerability to mental health symptoms. We also hypothesised perceived social support to protect against the negative effects of COVID-19 on maternal mental health.

Our results show that expectant women with higher depressive symptoms reported feeling less attached to their unborn baby. In line with previous studies demonstrating that women with higher depressive symptoms also have lower levels of maternal antenatal attachment (e.g. [12, 13, 38], we thus confirm previous observations that women’s mood during pregnancy influences the early relationship with her child. However, while anxiety symptoms and the psychological impact of the COVID-19 pandemic were positively associated to depression scores, in our study these measures did not predict the quality of maternal antenatal attachment. This is not entirely surprising, as the existent literature on the relationship between anxiety and antenatal attachment is mixed, with only some studies reporting a negative association between these two variables (e.g. [13, 16, 17].

The lack of association between stress related to the psychological impact of COVID-19 and antenatal attachment is similar to the findings reported by [39] that war trauma is not directly associated to maternal-fetal attachment in women living in war conditions. Similarly, other evidence suggests that other traumatic events such as history of prenatal loss are not associated to attachment to the fetus [40]. Nonetheless, the negative consequences of PTSD in the antenatal period are well documented in the literature. Stress during pregnancy negatively impacts on fetal brain development [41] as well as on children developmental outcomes (for a review see [42]. Furthermore, PTSD symptoms developed during pregnancy are associated to impaired maternal bonding to the infant at 6 weeks postpartum [43]. Therefore, it is likely that while PTSD in women pregnant during the COVID-19 pandemic did not affect the immediate relationship with their unborn baby, its negative impact could still manifest on postnatal attachment and on child development.

Our study also aimed to examine whether perceived social support and COVID-19 appraisal predicted mental health symptoms of anxiety and depression. We found that women who perceived the impact of COVID-19 to be more negative reported higher levels of anxiety (but not of depression) during their pregnancy. Previous research has demonstrated that increased stress can exacerbate symptoms of depression and anxiety during the perinatal period [44] and more recently, studies on the psychosocial impact of the COVID-19 pandemic have reported a surge in prenatal psychological distress among pregnant women [45–48]. However, our study also revealed that increased social support from partner, family and friends, and the healthcare system were associated with lower anxiety, suggesting that social support may protect against the negative effects of traumatic events, such as COVID-19. These findings extend previous studies on the importance of support from family, spouse, and other significant people for pregnant women [21, 49] as well as those from recent work [39] on the role of support in pregnant women living in war conditions, suggesting that support not only plays a critical role in life-endangering conditions of war, but also in any situation deemed traumatic.

It is also well established that poor social support is linked to vulnerability to depression and anxiety disorders [50, 51]. The restrictions imposed to control transmission of the virus and to manage the impact of changes to the NHS service have significantly altered the antenatal care of pregnant women, such as being unable to attend antenatal scans with a support person or seeing routine face-to-face appointments with midwives being replaced by telephone calls. These modifications to the maternity service during the COVID-19 pandemic led to changes in the support net usually available to women throughout their pregnancy. Our data show that, reduction in the perceived social support – as also confirmed by Fallon et al. [52]’s study - may have amplified anxiety symptoms, particularly for those women who appraised the impact of COVID-19 to be more negative.

Previous research has demonstrated that frequent support from pregnancy to the postnatal period, such as the one provided through continuity of care, weakens the association between high levels of stress experienced by women in the context of a natural disaster and mental health symptoms [53, 54]. Similarly, recent research on COVID-19 and mental health in Canadian expectant women, suggested that increased social support may buffer the negative effects of prenatal trauma [27]. In line with this literature, our findings point to the necessity of prioritising support for all pregnant women in order to protect their health and their infants’ developmental outcomes. Given the pivotal role that public health practitioners play in prevention, early identification and intervention for parents and their babies, it is necessary to provide health professionals with the appropriate tools and support to assess mental health difficulties throughout pregnancy, labour, birth, and the postnatal period.

Overall, our research shows that COVID-19 has affected the mental health of UK expectant women, with about 40-60% of them reporting symptoms of depression, anxiety and post-traumatic stress disorder. Given that the usual reported incidence of depression and anxiety during pregnancy are around 17% and 23%, respectively [5, 6], our study shows the extent to which COVID-19 has impacted on expectant women’s mental health. More direct converging evidence supporting the effect of the pandemic on pregnant women are also shown in our data included in the Supplementary Material. Specifically, we indicate that depressive symptoms in the group of women expecting a baby during the second UK lockdown were significantly higher compared to women that were pregnant before the COVID-19 pandemic (see Supplementary Material). This, together with the finding that 40% of the women in our study reported symptoms associated with post-traumatic stress disorder due to the pandemic, points towards a COVID-19-specific negative effect on prenatal maternal mental wellbeing.

It is also worth noting that, participants with a history of poor mental health reported higher depressive symptoms during pregnancy compared to participants that did not report previous mental health problems. In addition, those with high-risk pregnancies scored higher in prenatal anxiety and depression (see Supplementary Material). Prior history of poor mental health poses a significant risk for postnatal depression [55], and women with high-risk pregnancies are more likely to display concerning levels of prenatal anxiety and depression [56], although these rates don’t seem to have increased during the COVID-19 pandemic [57]. Our data adds to the existing literature by showing that prior history of poor mental health risk may significantly impact prenatal maternal mental wellbeing, especially in high-risk pregnant women.

Importantly, a recent study that looked at maternal postnatal mental health during the COVID-19 pandemic showed that 43 and 61% of women self-reported symptoms associated with clinically relevant depression and anxiety, respectively [52]. It is striking that in our study we find very similar high prevalence rates of depression (47%) and anxiety (60%) during pregnancy. We believe that the present evidence highlights two distinct needs that must be addressed in the near future; 1) mothers are facing a mental health crisis that needs urgent attention from authorities; 2) the cross-sectional nature of our study design prevents from drawing causal links among the variables of interest. Longitudinal research is therefore essential in examining the long-term impact of the pandemic and the relationship between prenatal and postnatal mental health, and later child outcomes.

Our research also presents other limitations. One of these is that we collected data only from mothers-to-be. Research has suggested that paternal perinatal mental health symptoms can exacerbate maternal symptoms [58] and predict behaviour problems in children when they are older [59, 60]. Thus, including assessment and preventing paternal prenatal mental health problems will be essential for future studies, and will offer the potential of maximising support for women and their children. Finally, our study was run prior to the vaccination programme in the UK. It would be important for future work to examine whether receiving a COVID-19 vaccine reduces COVID-19-specific negative effect on prenatal maternal mental wellbeing.

## Conclusions

Our research emphasises the need for heightened support from the beginning of pregnancy and for all mothers-to-be. The high rates of women reporting mental health symptoms of depression and anxiety during the COVID-19 pandemic highlighted by our study suggest that expectant women have been increasingly facing mental health difficulties that significantly interfere and impair the formation of a positive relationship with their unborn baby, and can potentially impact on childbirth outcome as well as infant and child long-term development. Importantly, we show that direct experience of prenatal trauma, such as the one experienced during the COVID-19 pandemic, can significantly amplify vulnerability to these mental health issues. Nonetheless, our study also highlights the protective role of social support on negative mental health outcomes, thus informing about intervention strategies that can protect maternal wellbeing during pregnancy and beyond.

## Supplementary Information

**Additional file 1.**

## Data Availability

Data and analysis code are available in the Open Science Framework "OSF": https://osf.io/kqjsn/.
