# Supplementary material for: The mental health crisis of expectant women in the UK: effects of the COVID-19 pandemic on prenatal mental health, antenatal attachment and social support
Source: BMC Pregnancy Childbirth. 2022 Jan 26;22:68. doi: 10.1186/s12884-022-04387-7 (PMC8790719; doi:10.1186/s12884-022-04387-7)
Supplement: Supplementary file 1 — Additional file 1. [file 12884_2022_4387_MOESM1_ESM.docx]

*Supplementary Material: “The mental health crisis of expectant women: effects of the COVID-19 pandemic* *on prenatal mental health, antenatal attachment and social support”*

**Maternal antenatal depressive symptoms pre- and post-COVID-19 pandemic**

We compared means of the BDI-II scores collected in the current study with data from unpublished research conducted by the senior member of the authorship team before the COVID-19 pandemic. The study was conducted online between September 2017 and November 2019, recruited expectant women with similar sample characteristics as the current research (see Table S1), and administered the BDI-II alongside a battery of other validated measures. Ethical approval was gained from the University of Essex Science and Health Ethics Sub-committee (SR1701).

We analysed depression scores (BDI-II) of three independent groups of expectant women; group 1 (N = 75; pre-COVID group henceforth) was tested before COVID-19 (September 2017 - November 2019), group 2 (N = 72; lockdown 1 group henceforth) was tested during the first UK lockdown (April 2020 – August 2020), and group 3 (N = 70; lockdown 2 group henceforth) was tested during the second UK lockdown (November 2020 – January 2021) (see Figure S1). Since the between-participant variance was not equal (as assessed by Levene’s Test), we report the values adjusted for equal variance not assumed.

Independent sample t-tests showed that, albeit the mean BDI-II scores were higher for the lockdown 1 group (M = 13.657, SD = 8.177) compared to the pre-COVID group (M = 11.480, SD = 5.381), there was no significant difference in scores between the two groups, *t*(111.993) = 1.850, *p* = 0.067. Similarly, there were no significant differences in BDI-II scores between the lockdown 1 group and lockdown 2 group (M = 15.88, SD = 9.673), *t*(134) = -1.45, *p* = 0.150. The difference in BDI-II scores between pre-COVID group and lockdown 2 group was significant, *t*(104.449) = 3.337, *p* = 0.001, suggesting that depression scores in the group of women expecting a baby during the second lockdown were significantly higher than the depression scores of women that were pregnant before the COVID-19 pandemic.

Table S1 Participants characteristics and pregnancy information for the sample of expectant women whose data was collected before the COVID-19 pandemic.

| **Participant characteristic** | | **Participant characteristic** | |
| --- | --- | --- | --- |
| *Age (mean year ± SD)* | 32.71 (3.83) | *Household size (including participant) (N/%)* | |
| *Years in education (mean year ± SD)* | 18.1 (2.64) | 1 person | 1 (1) |
| *Ethnicity (N/%)* |  | 2 people | 49 (69) |
| White | 62 (87) | 3 people | 17 (25) |
| Mixed/Multiple Ethnic Group | 5 (7) | 4 people | 4 (6) |
| Asian | 3(4) | **Pregnancy information** | |
| Hispanic | 1(1) | High risk pregnancy | 14 (20) |
| *Current relationship (N/%)* |  | Low risk pregnancy | 57 (80) |
| Married/civil partnership | 54 (76) |  |  |
| Cohabiting | 15 (20) |  |  |
| Single | 1(1) |  |  |
| Separated/divorced | 1 (1) |  |  |
|  |  |  |  |


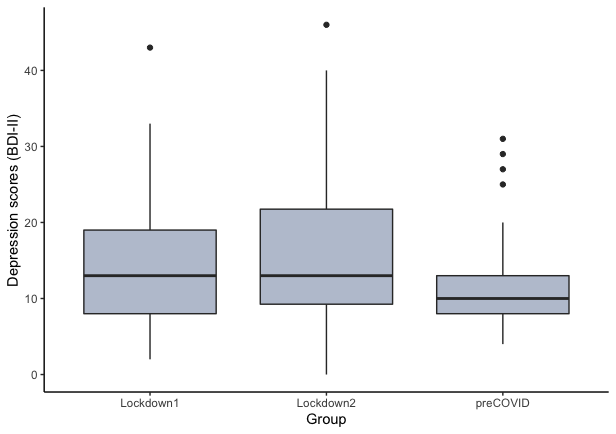


Figure S1. Bar plot showing mean depression scores across the three groups. The mean depression score of expectant women during lockdown 2 was significantly higher than the mean depression score of expectant women pre-COVID-19. There was no difference in depression scores between the two groups of women that were pregnant during the UK lockdown (lockdown 1 versus lockdown 2 groups) and between the pre-COVID-19 group and the lockdown 1 group.

**Maternal antenatal mental health and its association with pregnancy risk, mental health history, and pregnancy trimester**

We conducted univariate analysis of variance (one-way ANOVA) to examine whether history of mental health issues (as reported by participants) prior to the pregnancy could contribute to self-reported depression and anxiety during pregnancy. We found that, while depression was more likely to be reported in women with a history of mental health issues, F(2,133) = 8.837, *p* < .001, anxiety was not, F(2,145) = 1.050, *p* = .353.

Next, we examined whether pregnancy risk (high vs low risk) and trimester of pregnancy when the survey was completed (first vs second vs third trimester) could contribute to self-reported depression and anxiety during pregnancy. We found a main effect of pregnancy risk in both depression and anxiety scores. Specifically, depression and anxiety were more likely to be reported in women with high-risk pregnancies, depression: F(5,129) = 6.534, *p = .012*; anxiety: F(5,139) = 4.692, *p = .032*. We did not find a main effect of pregnancy trimester on either depression nor anxiety scores, depression: F(5,129) = 1.392, *p = .252*; anxiety: F(5,139) = .795, *p = .454*. We also did not find an interaction effect between pregnancy trimester and risk of pregnancy on the mental health scores, depression: F(5,129) = .499, *p* = .608; anxiety: F(5,139) = .546, *p = .581*.

**Sample size calculation**


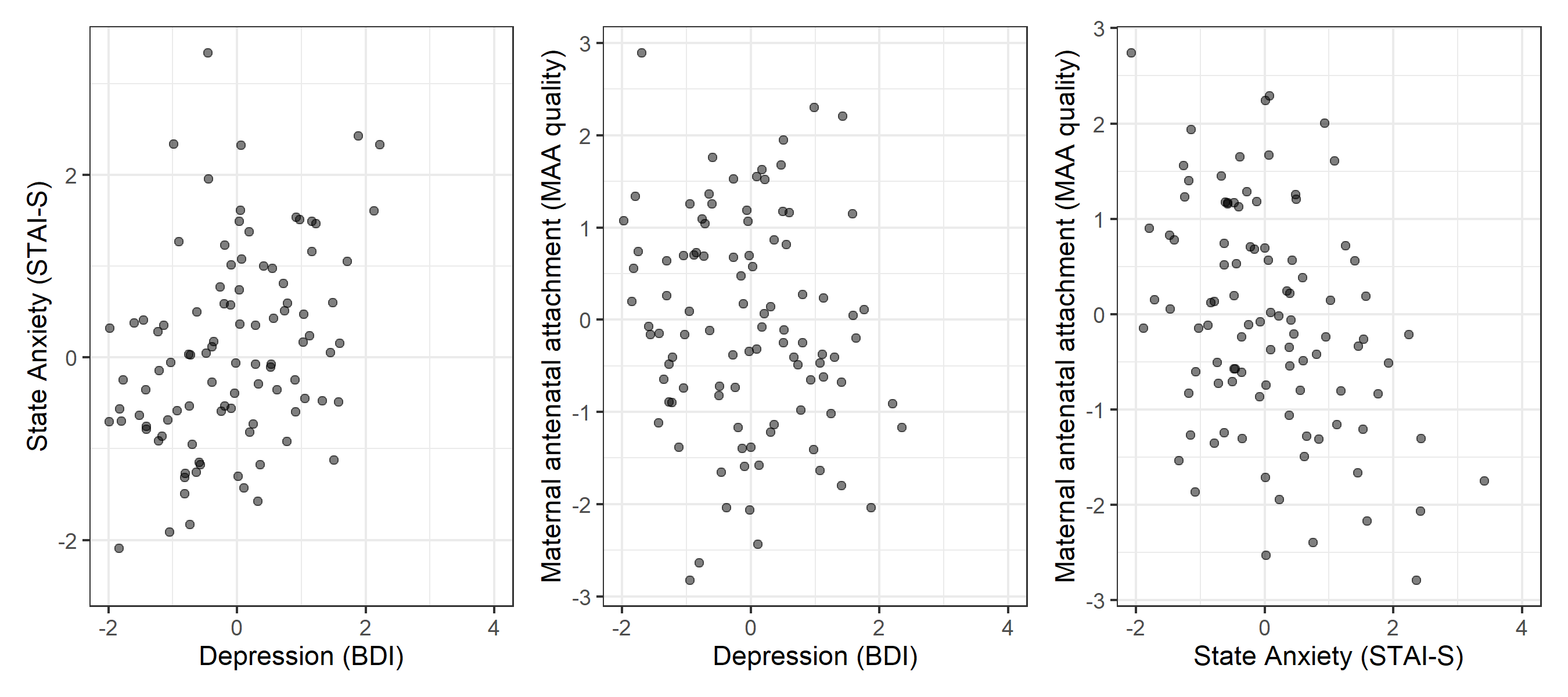


Simulated data for the effect of depression and anxiety on the quality of antenatal attachment. Each dot indicates a simulated participant.


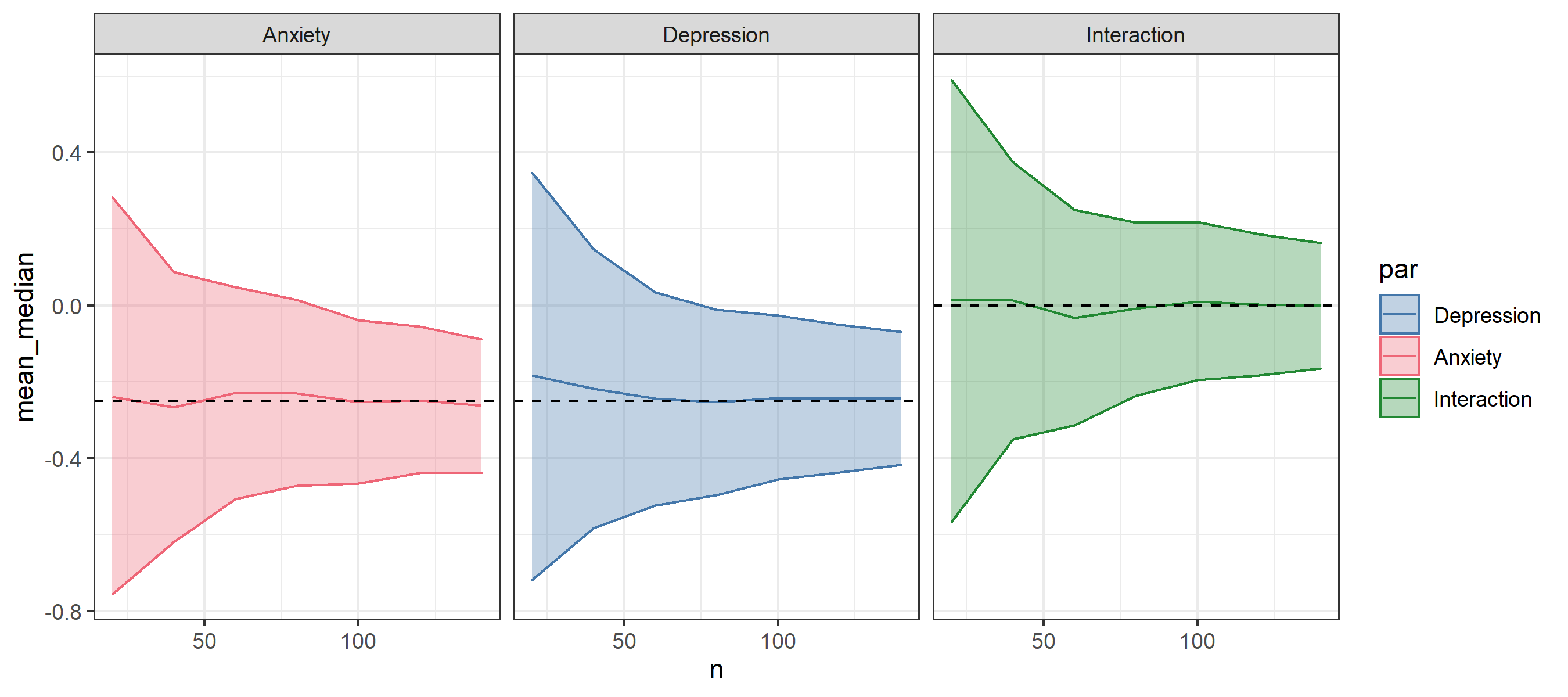


Figure 2.3: Sensitivity analysis: the effect of increasing the number of participants on the 95% HPDI estimates. We can see that sample sizes larger than n = 100 are sufficient to reliably detect that our simulated effect is < 0. Dashed lined indicates the simulated groundtruth.
